# Incidence and sociodemographic, living environment and maternal health associations with stillbirth in a tertiary healthcare setting in Kano, Northern Nigeria

**DOI:** 10.1186/s12884-022-04971-x

**Published:** 2022-09-08

**Authors:** Rebecca Milton, F. Modibbo, D. Gillespie, F. I. Alkali, A. S. Mukaddas, A. Kassim, F. H. Sa’ad, F. M. Tukur, R. Y. Khalid, M. Y. Muhammad, M. Bello, C. P. Edwin, E. Ogudo, K. C. Iregbu, L. Jones, K. Hood, P. Ghazal, J. Sanders, B. Hassan, F. J. Belga, T. R. Walsh

**Affiliations:** 1 Centre for Trials Research, Cardiff University, Cardiff, UK; 2 Murtala Mohammed Specialist Hospital (MMSH), Kano, Nigeria; 3 Department of Biochemistry, Bayero University, Kano, Nigeria; 4 Department of Biological Sciences, Bayero University, Kano, Nigeria; 5 Department of Medical Microbiology and Parasitology, Bayero University, Kano, Nigeria; 6 Department of Microbiology, Aminu Kano Teaching Hospital, Kano, Nigeria; 7 Department of Medical Microbiology, National Hospital Abuja, Abuja, Nigeria; 8 Department of Medical Microbiology Cardiff, Public Health Wales, University Hospital of Wales, Cardiff, UK; 9 Systems Immunity Research Institute, School of Medicine, Cardiff University, Cardiff, UK; 10 School of Healthcare Sciences, Cardiff University, Cardiff, UK; 11 Institute of Infection and Immunity, School of Medicine, Cardiff University, Cardiff, UK; 12 Department of Zoology, Ineos Institute of Antimicrobial Research, University of Oxford, Oxford, UK

**Keywords:** Stillbirth, Sub-Saharan Africa, Nigeria, Mothers, Preventable mortality, Global health

## Abstract

**Background:**

Almost two million stillbirths occur annually, most occurring in low- and middle-income countries. Nigeria is reported to have one of the highest stillbirth rates on the African continent. The aim was to identify sociodemographic, living environment, and health status factors associated with stillbirth and determine the associations between pregnancy and birth factors and stillbirth in the Murtala Mohammed Specialist Hospital, Kano, Nigeria.

**Methods:**

A three-month single-site prospective observational feasibility study. Demographic and clinical data were collected. We fitted bivariable and multivariable models for stillbirth (yes/no) and three-category livebirth/macerated stillbirth/non-macerated stillbirth outcomes to explore their association with demographic and clinical factors.

**Findings:**

1,998 neonates and 1,926 mothers were enrolled. Higher odds of stillbirth were associated with low-levels of maternal education, a further distance to travel to the hospital, living in a shack, maternal hypertension, previous stillbirth, birthing complications, increased duration of labour, antepartum haemorrhage, prolonged or obstructed labour, vaginal breech delivery, emergency caesarean-section, and signs of trauma to the neonate following birth.

**Interpretation:**

This work has obtained data on some factors influencing stillbirth. This in turn will facilitate the development of improved public health interventions to reduce preventable deaths and to progress maternal health within this site.

**Supplementary Information:**

The online version contains supplementary material available at 10.1186/s12884-022-04971-x.

## Background

The World Health Organization (WHO) defines a stillbirth as ‘a baby born with no signs of life at or after 28 weeks' gestation’ [1]. Although the WHO definition is used for international comparison, the definition of stillbirth varies between countries with the reference time points ranging between 20- and 28-weeks’ gestation [2]. Stillbirths are reported as one of the most neglected tragedies in global health today, with almost two million stillbirths occurring each year and the majority occurring in low- and middle-income countries (LMICs) [3, 4]. Three quarters of global stillbirths are reported to occur in sub-Saharan Africa (SSA) and south Asia [5]. Antenatal stillbirths are often due to preventable conditions such as maternal infections and non-communicable diseases [3, 4, 6]. Almost half of all stillbirths occur during the intrapartum period, with many linked to obstetric complications [3, 4], in contrast to high-income countries (HICs) where intrapartum stillbirths are rare [7]. Other known risk factors for stillbirths overall include young or advancing maternal age, fetal infection, maternal hypertensive conditions, perinatal asphyxia, history of previous stillbirth, obstetric complications such as intrauterine growth restriction and abruptio placenta and placenta praevia [8–10]. Common non-clinical risk factors include lack of maternal education, socioeconomic deprivation and substandard antenatal care [10].

Stillbirths are commonly measured as rates per 1000 births; UNICEF reports that the estimated stillbirth rate in SSA is 21.7/1000; compared to 2.9/1000 births in Western Europe, making mothers in LMICs seven times more likely to deliver a stillborn baby than their counterparts in HICs [5]. Nigeria is reported to have one of the highest stillbirth rates on the African continent and is one of six countries that bear the burden of 50% of global stillbirths [3]. In addition to these data it is reasonable to assume that the reported rates are an underestimation due to the cultural challenges faced in LMICs surrounding the reporting of stillbirths [11]. Obtaining reliable and accurate data requires known gestational age at birth, a clear definition of a stillbirth, reliable reporting systems both in-facility and communities, an increase in facility-based births and a reduction of unattended homebirths; many of these aspects are underdeveloped in LMICs [12].

This study was conducted in the Murtala Mohammed Specialist Hospital (MMSH), a tertiary hospital located in northern Nigeria, serving a population of approximately 11 million and where stillbirths were previously not statutorily registered. Preliminary observational work carried out at the MMSH, found the incidence of stillbirth to be 180/1000 births [13]. The work reported in this manuscript was conducted as a component part of a wider feasibility study ‘Stillbirths in Kano’, with the primary study objective being to identify whether stillborn babies in MMSH can be classified using an established system via qualitative, clinical, epidemiology, microbiology, and immunology methods. A key output of the feasibility study was to inform further research and pave the way for future intervention implementation to prevent stillbirth using a mixed-methods approach. The primary aims of the quantitative component, covered in this manuscript, were to identify the sociodemographic, living environment, and health status factors associated with stillbirth, and determine the associations between birth and pregnancy factors and stillbirth in MMSH, Kano, Nigeria. The secondary aims were to identify how well sociodemographic factors predict stillbirth and what is the improvement in predictive performance when including i.) living environment, ii.) health and medical history and iii.) pregnancy history factors; as well as examining at whether the associations between sociodemographic, living environment, and health status factors and stillbirth are consistent across macerated or non-macerated stillbirths; this being the marker for antenatal or intrapartum death of the fetus.

## Methods

### Study design

A single site prospective observational study conducted over a three-month period.

### Setting

The MMSH, a tertiary referral hospital located in Kano, Nigeria. There are 1000–1200 general hospital beds, 17 neonatal intensive care unit (NICU) beds, 133 maternity beds and 22 delivery cubicles. Every month there are approximately 550 deliveries with four midwives on each shift, two allocated to complicated deliveries and two for uncomplicated deliveries. The MMSH is a multi-disciplinary hospital and used as a referral hospital by many surrounding states and some neighbouring countries, contributing to the hospital being extremely under-resourced. The MMSH is owned by the state Government, and it offers free services to its patients, regardless of where the patient is from.

### Participants

Mothers presenting at the MMSH in labour and their neonates, between October 2018 and January 2019.

### Eligibility criteria

Any mother presenting at the MMSH in labour with intent to deliver in the MMSH within the study period and who provided informed consent was eligible for this research study.

### Ethics and informed consent

This research was performed in accordance with the Declaration of Helsinki and ethical approval was sought and given by Health Research Ethics Committee, Kano State of Nigeria Ministry of Health REF: MOH/Off/797/T.1/950 on the 4^th^ of September 2018. Mothers were provided with study information in their local dialect and written informed consent was obtained by trained research nurses.

### Procedures

Recruitment of mothers was conducted consecutively, there was a short period where recruitment paused over Christmas due to excessive staff shortages. Demographic and clinical data were collected by paper-based questionnaires by trained research nurses. Data were collected on living environment, health and medical history, pregnancy history and pregnancy and birth factors. Each mother answered 24 pre-delivery questions, with seven potential follow-on questions dependent on birth outcome. A further 21 and 28 data points were collected from clinical observations for live and stillborn neonates respectively, with four potential follow-on questions (Supplementary file P1-11). Photographs were taken of stillborn babies to support data collected and to aid RM, TW, DG and LJ from Cardiff University on classifying degrees of maceration in an attempt to identify antenatal and intrapartum fetal death. Macerated stillbirths were defined as those with signs of maceration at delivery including skin and soft-tissue changes such as skin discoloration, redness, sloughing of skin, necrosis and severe overriding of cranial bones.

### Statistical methods

Descriptive statistics were reported as frequencies and percentages, means and standard deviations, and medians and interquartile ranges as appropriate. Bivariable associations between demographic and clinical variable for the outcome stillbirth (yes/no) were investigated by fitting binary logistic regression models. Findings were reported as odds ratios (OR), 95% confidence intervals (CI), and *p*-values.

Multivariable models explored the adjusted association between demographic and clinical variables and stillbirth. Models were built up in stages, with each new stage building on previous stages (i.) sociodemographic factors; ii.) living environment; iii.) mother’s health and medical history; iv.) pregnancy history). The ordering enabled an understanding as to which variables were potentially more amenable to proactive intervention (e.g., health education, sanitation). Data on these earlier variables are generally easier to obtain from methods as they are easier to recall accurately. We sought to further explore maternal medical history and pregnancy history to build a more complete picture and to understand which factors were associated with stillbirth. Model data are reported as adjusted OR, 95% CI, and *p*-values. For each model, the AUROC and pseudo R^2^ statistics (both Cox and Snell and Nagelkerke's R^2^) are also reported.

Exploratory associations between demographic, clinical data, and stillbirth sub-divided into macerated or non-macerated were investigated by fitting multinomial logistic regression models, with livebirth as the base outcome. Findings were reported as relative risk ratios (RRR), 95% CI, and p-values. We also conducted exploratory analyses, separate from the modelling approach describe above, to investigate the associations between birth factors and stillbirth (binary logistic regression) and type of stillbirth (multinomial logistic regression). Continuous variables were investigated for non-linearity by comparing linear fits to polynomials and restricted cubic spline terms [14]. Owing to the small amount of missing data, models were based on complete cases. Statistical analyses were conducted using Stata v16.1.

### Role of the funding source

The funder had no role in study design; in the collection, analysis, and interpretation of data; in the writing of the manuscript; nor in the decision to submit the paper for publication. The corresponding author confirms that she had full access to all the data in the study and had final responsibility for the decision to submit for publication.

## Results

This study included 1,998 neonates born to 1,926 mothers who consented to participate in the study. Most variables were obtained from all participants; and complete data were available for 1,989 births (99.5%).

Most mothers (63.4% *n* = 1222) were aged between 20 and 30 years, with only 2.6% (*n* = 51) of mothers aged over 40. The lower three tiers of household income were evenly distributed, around 30% of mothers in each tier with 11.1% (*n* = 215) in the upper two tiers of household income. The largest proportion of mothers reported living in a house (47.1% *n* = 907) with 30.2% (*n* = 581) living in an apartment, in this area an apartment would be the preferred choice, and 23% (*n* = 437) living in a shack. Over half of mothers had at least secondary school level education (61.7% *n* = 1,188), with 22.8% (*n* = 440) reporting to have no formal education and 15.5% (*n* = 298) educated to primary school level. The employment distribution was relatively even with 44.7% (*n* = 860) employed and 55.3% (*n* = 1066) unemployed. There was diversity among household access to water; water vendors supplied 33% (*n* = 636), 29.3% (*n* = 565) had access to a private well, 19% (*n *= 366) accessed water via a municipal network, e.g., home has water via a networked tap, this is the preferred option, 13% (*n* = 250) via communal taps and 5.7% (*n* = 109) via private boreholes. Most mothers had access to a sit/squat toilet with flush within the house (68.4% *n* = 1317), and 31.4% (*n *= 604) had access to a pit latrine (Supplementary table 1, supplementary file P12-14).

Most mothers had previously been pregnant 76.6% (*n* = 1476) and of the total cohort, 15.1% (*n* = 290) had previously experienced a stillbirth. Grand-multiparty is defined in this setting as having five or more children, 36.2% (*n* = 698) mothers were considered to have grandmultiparity. Most mothers reported having a healthy weight (89% *n* = 1715), 73.9% (*n* = 1424) reported taking some medication, antibiotic use was reported in 7.8% (*n* = 150) mothers and 28.1% (*n* = 542) reported taking antimalarial medication. Throughout pregnancy few mothers reported taking vitamins 8.1% (*n* = 150), with 40.4% (*n* = 779) taking folic acid/iron/platelets supplements and 55.1% (*n=*1062) reported taking pain relief. More than half (53.8% *n* = 1036) reported a health condition, 29.6% (*n* = 571) reported having malaria and 9.8% (*n* = 188) reported hypertension (Supplementary table 1, supplementary file P12-14).

Over half lived within 10 kilometres (km) of the hospital (58.4% *n* = 1125), as the distance increased the percentage of mothers decreased, yet some (0.8% *n* = 16) mothers travelled over 100 km to be admitted to the MMSH (Supplementary table 1, supplementary file P12-14).

Most mothers had at least one ultrasound during pregnancy (73.7% *n* = 1420) and 93.9% (*n* = 1808) noticed regular fetal movement in the prior 24 h to delivery. Multiple pregnancies made up 3.6% (*n* = 69) of the cohort. The most frequently observed mode of delivery was spontaneous vaginal delivery (SVD) (82.4% *n* = 1646), 15.5% (*n* = 309) were caesarean-sections, 2.1% (*n* = 42) deliveries were reported as vaginal breech. The most common presentation was cephalic; 88.4% (*n* = 1767), 9.3% (*n* = 185) breech presentation, 1.2% (*n *= 23) were compound presentation, 0.7% (*n* = 13) face presentation and 0.5% (*n* = 10) shoulder presentation. Signs of trauma were identified in 5% (*n* = 100) of neonates and 3.6% (*n *= 69) were part of a multiple birth (Supplementary table 1, supplementary file P12-14).

Of the 1998 births, 1789 were livebirths and 209 were stillbirths. Of the stillborn babies 100 had signs of maceration and 109 had no signs. The stillbirth rate within this cohort was 105/1000 births.

Bivariable models found associations between increased maternal age and stillbirth (Table 1). The mulitvariable models found that the association remained only within age categories 25–30 years (OR: 1.65, 95% CI: 1.07 to 2.55, *p* = 0.024) and 31–35 years (OR: 1.76, 95% CI: 1.04 to 3.00, *p* = 0.037) after adjusting for demographics, living environment and health and medical history (Table 2). After adjusting for pregnancy history, no evidence of an association was found between maternal age and stillbirth (Table 2). Bivariable models found that mothers educated up to primary school level had higher odds of stillbirth compared to those who had secondary school or above level of education; (OR 3.40, 95% CI: 2.52 to 4.59, *p* < 0.001) (Table 1), this finding remained after adjusting for all sociodemographic and health features (Table 2). No evidence of an association was found between stillbirth and household monthly income or employment status (Table 1). The pseudo r^2^ for the multivariable model including demographic features was 0.0562 (Supplementary table 2, supplementary file P15).

**Table 1:** Bivariable associations between sociodemographic, living environment, maternal health and medical history and pregnancy history variables and stillbirth

Domain	Variable	Categories	OR (95% CI)	*p*-value
Demographics	Mothers age (years)	< 20	1.34 (0.73 to 2.47)	0.349
		20–24	Reference category	
		25–30	1.96 (1.31 to 2.95)	0.001
		31–35	2.19 (1.35 to 3.57)	0.002
		36–40	2.34 (1.41 to 3.89)	0.001
		> 40	3.68 (1.76 to 7.72)	0.001
	Monthly household income (NGN)	45,001 or higher	Reference category	
		Up to 45,000	1.22 (0.90 to 1.65)	0.195
	Employment status	Employed	Reference category	
		Unemployed	1.07 (0.80 to 1.43)	0.633
	Highest level of education	Secondary school or above	Reference category	
		None or primary school	3.40 (2.52 to 4.59)	< 0.001
Living environment	Distance travelled to the hospital	< 10 km	Reference category	
		10-30 km	1.57 (1.15 to 2.14)	0.005
		31-50 km	5.43 (3.14 to 9.39)	< 0.001
		51-100 km	5.18 (2.08 to 12.92)	< 0.001
		> 100 km	5.38 (1.83 to 15.83)	0.002
	Type of residence	Apartment or house	Reference category	
		Shack or other	2.59 (1.92 to 3.49)	< 0.001
	Primary household water source	Municipal network	Reference category	
		Not municipal network	1.90 (1.22 to 2.96)	0.004
	Household toilet facilities	Toilet with flush	Reference category	
		Pit latrine or other	2.94 (2.20 to 3.94)	< 0.001
Health and medical history	Self-perceived nutritional status	Healthy Weight	Reference category	
		Overweight	0.97 (0.51 to 1.84)	0.918
		Underweight	1.97 (1.19 to 3.28)	0.009
	Medication	Antibiotics	1.57 (0.98 to 2.50)	0.061
		Anti-inflammatories	2.15 (0.24 to 19.29)	0.496
		Pain relief	0.65 (0.49 to 0.87)	0.003
		Traditional	1.35 (0.92 to 1.98)	0.126
		Antiretroviral therapy (ART)	2.15 (0.24 to 19.29)	0.496
		Other	0.86 (0.46 to 1.64)	0.655
	Supplements	Vitamins	1.03 (0.61 to 1.74)	0.921
		Folic acid / iron / haemoglobin	0.65 (0.48 to 0.88)	0.006
	Health conditions	Malaria	0.66 (0.47 to 0.93)	0.018
		Fever / Infection	2.07 (0.99 to 4.35)	0.054
		Hypertension	1.88 (1.26 to 2.82)	0.002
Pregnancy History	Pregnancy history	Previous stillbirth	2.96 (2.14 to 4.08)	< 0.001
		Grand Multiparity (≥ 5 previous pregnancies)	2.43 (1.82 to 3.24)	< 0.001

**Table 2:** Multivariable associations between sociodemographic, living environment, maternal health and medical history and pregnancy history variables and stillbirth

Domain	Variable		Block 1		Block 2		Block 3		Block 4	
			**OR (95% CI)**	***p*-value**	**OR (95% CI)**	***p*-value**	**OR (95% CI)**	***p*-value**	**OR (95% CI)**	***p*-value**
Demographics	Mothers age	< 20	1.11 (0.59 to 2.06)	0.747	0.95 (0.50 to 1.81)	0.873	0.95 (0.49 to 1.83)	0.878	1.05 (0.55 to 2.03)	0.878
		20–24	Reference category							
		25–30	1.65 (1.08 to 2.51)	0.019	1.59 (1.04 to 2.44)	0.034	1.65 (1.07 to 2.55)	0.024	1.32 (0.82 to 2.11)	0.252
		31–35	1.60 (0.96 to 2.67)	0.072	1.72 (1.02 to 2.91)	0.041	1.76 (1.04 to 3.00)	0.037	1.21 (0.65 to 2.27)	0.550
		36–40	1.47 (0.86 to 2.53)	0.161	1.49 (0.85 to 2.60)	0.160	1.41 (0.80 to 2.50)	0.237	0.93 (0.48 to 1.83)	0.839
		> 40	1.97 (0.91 to 4.26)	0.085	2.25 (1.01 to 5.00)	0.047	2.20 (0.97 to 4.97)	0.058	1.26 (0.51 to 3.12)	0.614
	Household monthly income (NGN)	< 45,000	Reference category							
		≥ 45,000	1.07 (0.78 to 1.47)	0.686	0.96 (0.69 to 1.33)	0.807	0.91 (0.64 to 1.28)	0.574	0.92 (0.65 to 1.31)	0.658
	Employment status	Employed	Reference category							
		Unemployed	1.19 (0.87 to 1.62)	0.267	1.18 (0.86 to 1.62)	0.307	1.04 (0.75 to 1.46)	0.804	1.13 (0.81 to 1.59)	0.470
	Highest level of education	Secondary school or above	Reference category							
		None or primary school	3.04 ( 2.21 to 4.20)	< 0.001	2.23 (1.50 to 3.02)	< 0.001	2.12 (1.48 to 3.01)	< 0.001	1.94 (1.35 to 2.79)	< 0.001
Living environment	Distance travelled to hospital	< 10 km			Reference category					
		10-30 km			1.48 (1.07 to 2.04)	0.019	1.50 (1.08 to 2.10)	0.016	1.52 (1.08 to 2.12)	0.015
		31-50 km			3.56 (1.96 to 6.45)	< 0.001	3.82 (2.06 to 7.11)	< 0.001	3.64 (1.93 to 6.85)	< 0.001
		51-100 km			3.85 (1.48 to 10.03)	0.006	3.23 (1.19 to 8.72)	0.021	3.39 (1.25 to 9.21)	0.017
		> 100 km			4.57 (1.41 to 14.87)	0.012	4.14 (1.25 to 13.65)	0.020	4.44 (1.31 to 15.05)	0.017
	Type of residence	Apartment or house			Reference category					
		Shack or other			1.50 (1.07 to 2.10)	0.018	1.55 (1.09 to 2.20)	0.014	1.50 (1.05 to 2.13)	0.026
	Primary household water source	Municipal network			Reference category					
		Not municipal network			1.43 (0.90 to 2.26)	0.130	1.46 (0.91 to 2.32)	0.113	1.47 (0.92 to 2.35)	0.110
	Household toilet facilities	Toilet with flush			Reference category					
		Pit latrine or other			1.57 (1.11 to 2.21)	0.010	1.49 (1.05 to 2.11)	0.027	1.41 (0.99 to 2.02)	0.055
Health and medical history	Self-perceived nutritional status	Healthy weight					Reference category			
		Overweight					0.82 (0.42 to 1.63)	0.579	0.78 (0.39 to 1.57)	0.488
		Underweight					1.35 (0.77 to 2.38)	0.297	1.46 (0.83 to 2.59)	0.189
	Medication	Antibiotics					1.56 (0.91 to 2.68)	0.107	1.54 (0.89 to 2.66)	0.122
		Anti-inflammatories					0.78 (0.06 to 9.74)	0.850	1.05 (0.09 to 12.77)	0.968
		Pain relief					0.58 (0.39 to 0.85)	0.005	0.59 (0.40 to 0.87)	0.008
		Traditional					1.43 (0.92 to 2.24)	0.113	1.40 (0.89 to 2.21)	0.142
		Antiretroviral therapy (ART)					1.57 (0.15 to 16.47)	0.708	1.64 (0.15 to 18.13)	0.688
		Other					0.99 (0.50 to 1.96)	0.979	1.07 (0.54 to 2.12)	0.853
	Supplements	Vitamins					1.02 (0.57 to 1.82)	0.941	1.01 (0.57 to 1.81)	0.969
		Folic acid / iron / haemoglobin					0.94 (0.65 to 1.36)	0.748	0.93 (0.64 to 1.34)	0.685
	Health conditions	Malaria					0.78 (0.54 to 1.15)	0.212	0.76 (0.52 to 1.12)	0.169
		Fever / infection					2.48 (1.12 to 5.49)	0.025	2.53 (1.12 to 5.70)	0.025
		Hypertension					1.95 (1.24 to 3.09)	0.004	1.95 (1.23 to 3.10)	0.005
Pregnancy History	Pregnancy History	Grand multiparity ≥ 5 pregnancies							1.43 (0.91 to 2.23)	0.119
		Previous stillbirth(s)							2.37 (1.64 to 3.42)	< 0.001

In the bivariable model, a greater distance to travel from home to the hospital was associated with higher odds of stillbirth (Table 1); this association remained after adjusting for sociodemographic and health features (Table 2). Distances were compared to a reference category of < 10 kms (10-30 km: OR: 1.52 95% CI: 1.08 to 2.12 *p* = 0.015; 31-50 km: OR: 3.64 95% CI: 1.93 to 6.85 *p* < 0.001; 51-100 km: OR: 3.39 95% CI: 1.25 to 9.21 *p* = 0.017 and > 100 km: OR: 4.44 95% CI: 1.31 to 15.05 *p* = 0.017). Living in a shack compared to an apartment or a house was associated with higher odds of stillbirth (OR: 2.59 95% CI: 1.92 to 3.49 *p* < 0.001) (Table 1). After adjusting for all sociodemographic and health features in the multivariable model this risk remained (Table 2).

Compared to a toilet with a flush, a pit latrine as the household toilet facilities was associated with higher odds of stillbirth (OR: 2.94 95% CI: 2.20 to 3.94 *p *< 0.001) (Table 1), this association remained after adjusting for all sociodemographic and health features in the multivariable model (Table 2). If the primary source of water was not a municipal network the odds of stillbirth were higher (OR: 1.90 95% CI: 1.22 to 2.96 *p* = 0.004) in the bivariable analysis. However, this association did not remain after adjusting for sociodemographic and health features. The pseudo-r^2^ for the multivariable model including demographic and living environment features was 0.0992, representing an absolute increase from the initial model of 0.043 and a relative increase of 76.5% (Supplementary table 2, supplementary file P15).

Bivariable models found the following factors to be associated with higher odds of stillbirth: mother’s perceived nutritional status as underweight (OR: 1.97 95% CI: 1.19 to 3.28 *p* = 0.009) and hypertension (OR: 1.88 95% CI: 1.26 to 2.82 *p* = 0.002) (Table 1). When adjusting for all sociodemographic and health features hypertension remained associated with higher odds of stillbirth (OR: 1.95 95% CI: 1.23 to 3.10 *p* = 0.005) (Table 2). Having fever or infection was associated with stillbirth, only once sociodemographic and health features had been adjusted for (OR: 2.53 95% CI: 1.12 to 5.70 *p* = 0.025) (Table 2). No evidence of an association was found between stillbirth and maternal use of antibiotics, anti-inflammatories, traditional medicine, antiretroviral therapy, or vitamins, neither was being overweight (Table 2). The pseudo-r^2^ for the multivariable model include demographic and living environment features was 0.1223 representing an absolute increase from the initial model of 0.0231 and a relative increase of 23.3% (Supplementary table 2, supplementary file P15).

In both the bivariable and multivariable models, a previous stillbirth was associated with higher odds of a stillbirth, after adjusting for all sociodemographic and health features the odds of having a stillbirth were over double that of a first-time mother (OR: 2.37, 95% CI: 1.64 to 3.42, *p* = <0.001) (Tables 1 and 2). The pseudo-r^2^ for the multivariable model include demographic and living environment features was 0.1394 representing an absolute increase from the initial model of 0.0171 and a relative increase of 14% (Supplementary table 2, supplementary file P15).

The bivariable analysis conducted on pregnancy and birth related factors are shown in Table 3. Compared to cephalic presentation shoulder presentation, compound presentation and breech presentation were all associated with higher odds of stillbirth (Shoulder: OR: 17.17, 95% CI: 4.79 to 61.54, *p* < 0.001; Compound: OR: 8.80, 95% CI: 3.79 to 20.43, *p* < 0.001; Breech: OR: 4.12, 95% CI: 2.85 to 5.96, *p* < 0.001). Birthing complications were associated with increased odds of stillbirth (OR: 5.98, 95% CI: 4.43 to 8.07, *p* < 0.001).

**Table 3:** Bivariable analysis; associations with pregnancy and birth related factors and stillbirth

Domain	Variable	Categories	OR (95% CI)	*p*-value
Pregnancy / birth related	Singleton or multiple	Multiple pregnancy	0.51 (0.25 to 1.05)	0.068
	Ultrasound in pregnancy	Yes	0.74 (0.54 to 1.01)	0.054
	Presentation	Shoulder presentation	17.17 (4.79 to 61.54)	< 0.001
		Face presentation	2.08 (0.46 to 9.48)	0.344
		Compound presentation	8.80 (3.79 to 20.43)	< 0.001
		Cephalic presentation	Reference category	
		Breech presentation	4.12 (2.85 to 5.96)	< 0.001
	Birthing complications	Yes	5.98 (4.43 to 8.07)	< 0.001
	Duration of labour	< 18 h	Reference category	
		≥ 18 h	2.92 (2.10 to 4.07)	< 0.001
		Unknown	3.42 (1.59 to 7.36)	0.002
	Antepartum haemorrhage	Yes	6.66 (4.59 to 9.68)	< 0.001
	Delivery	Prolonged / obstructed labour	3.34 (1.96 to 5.69)	< 0.001
		Prolonged labour^a^	1.61 (1.18 to 2.20)	0.003
		Vaginal breech delivery	4.61 (2.34 to 9.05)	< 0.001
		SVD	Reference category	
		Emergency C-section	2.89 (2.02 to 4.15)	< 0.001
		Elective C-section	0.12 (0.02 to 0.87)	0.036
	Sex	Male	Reference category	
		Female	1.02 (0.76 to 1.36)	0.913
		Unable to determine	1.00	
	Gestational age (weeks)	GA	0.23 (0.11 to 0.49)	< 0.001
		GA (squared)	1.02 (1.01 to 1.03)	0.001
	Birth weight	BW spline term 1^b^	0.22 (0.16 to 0.30)	< 0.001
		BW spline term 2^b^	5.67 (3.59 to 8.96)	< 0.001
	Signs of birth trauma (neonate)	Yes	5.61 (3.62 to 8.68)	< 0.001

^a^adjusted due to parity

BW^b^modelled as a cubic spline with three knots (add details from PDF)

HC + modelled as a cubic spline with three knots (add details from PDF)

Duration of labour which was 18 h or greater or reported unknown in duration were both associated with increased odds of stillbirth (≥ 18 h: OR: 2.92, 95% CI: 2.10 to 4.07, *p* < 0.001; unknown: OR: 3.42, 95% CI: 1.59 to 7.36, *p* = 0.002). Prolonged or obstructed labour was associated with increased stillbirth odds (OR: 3.34, 95% CI: 1.96 to 5.69, *p* < 0.001). Prolonged labour was adjusted for parity and remained statistically significantly associated with increased odds (OR: 1.61, 95% CI: 1.18 to 2.20, *p* = 0.003). Antepartum haemorrhage was associated with increased odds of stillbirth (OR: 6.66, 95% CI: 4.59 to 9.68, *p* < 0.001) (Table 3).

Compared to spontaneous vaginal delivery, vaginal breech delivery and emergency caesarean section were both associated with increased odds of stillbirth (VBD: OR: 4.61, 95% CI: 2.34 to 9.05, *p* < 0.001; EmC: OR: 2.89, 95% CI: 2.02 to 4.15, *p* < 0.001).

**Fig. 1:**
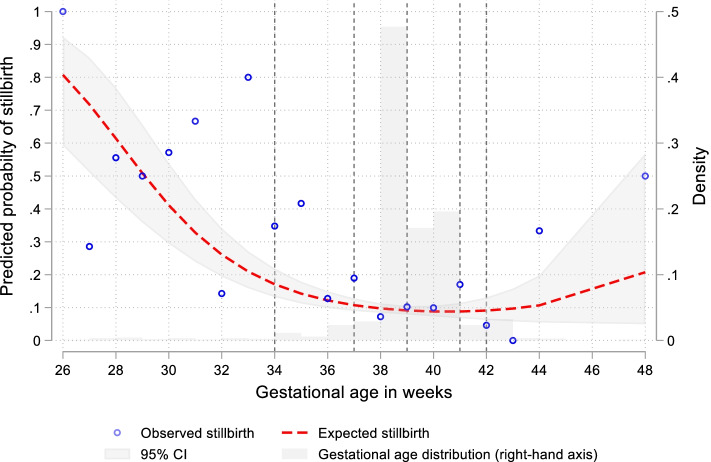
Association between gestational age and stillbirth

A non-linear association between gestational age and stillbirth was identified, with the odds of stillbirth decreasing as gestational age increased from 26 to 37 weeks, stable between 37 and 42 weeks and increasing again thereafter, with greater uncertainty at the extremes of our gestational ages (*p*-value for joint test of linear and quadratic term < 0.001) (Fig. 1). Similarly with birthweight and stillbirth a non-linear association was found, with the odds of stillbirth decreasing as birthweight increased from 0.5 kg to 3.0 kg, stable between 3.0 kg and 3.5 kg and increasing again thereafter (Supplementary Fig. 1, supplementary file P16).

**Fig. 2:**
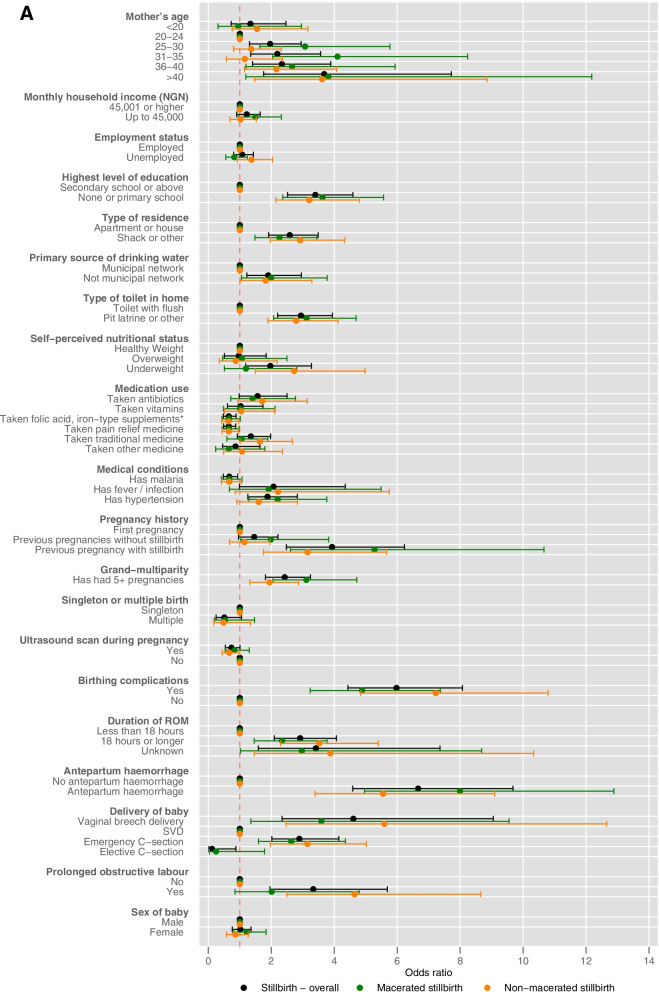

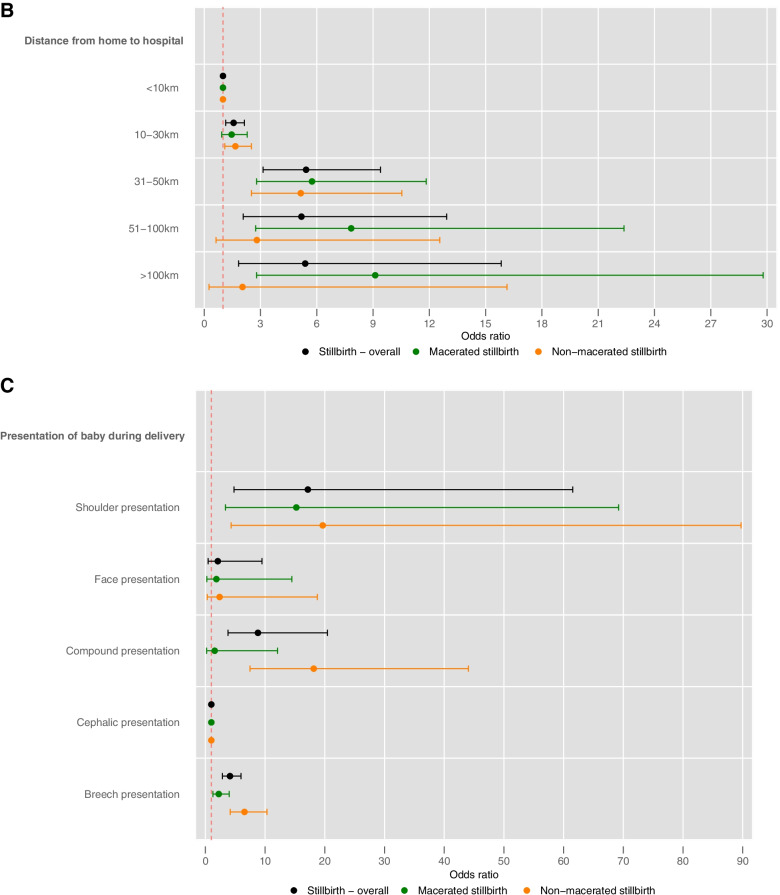
a, b, c (top to bottom): Forest Plots showing associations with **a**.) sociodemographic, living environment, maternal health and medical history and pregnancy history, pregnancy and birth related, **b**.) distance from home to hospital, **c**.) presentation of baby during delivery and stillbirth overall, macerated stillbirth and non-macerated stillbirth *Includes platelet and haemoglobin supplements

Most associations were consistent with the binary stillbirth analysis (Supplementary table 3, supplementary file P17-19). However, there were some differences in statistically significant associations once divided into stillbirth classifications. The use of traditional medicine in the three months prior to birth was associated with a higher risk of non-macerated stillbirth (RR: 1.64, 95% CI: 1.01 to 2.67, *p *= 0.047), in the binary stillbirth analysis the use of traditional medicines and stillbirth were not statistically significantly associated. In the multinomial stillbirth analysis, being underweight was associated with a higher risk of non-macerated stillbirth only (RR: 2.73, 95% CI: 1.50 to 4.98, *p* = 0.001). The use of folic acid or iron supplements was associated with a lower risk of non-macerated stillbirth (RR: 0.64, 95% CI 0.42 to 0.97, *p* = 0.033). Across both stillbirth classifications malaria was no longer statistically significant. Hypertension was only associated with a higher risk of macerated stillbirths (RR: 2.20, 95% CI 1.29 to 3.76, *p* = 0.004). Having at least one ultrasound during pregnancy was associated with a lower risk of non-macerated stillbirth (RR: 0.66, 95% CI: 0.44 to 1.00, *p* = 0.049) whereas in the binary stillbirth analysis no association was found. The binary stillbirth analysis found that prolonged or obstructed labour was associated with a higher risk of stillbirth, yet within the multinomial stillbirth analysis the association was only statistically significant within non-macerated stillbirth (RR: 4.65, 95% CI: 2.49 to 8.65, *p *< 0.001). Compound presentation was found to be associated with non-macerated stillbirth (RR: 18.15, 95% CI: 7.47 to 44.05, *p* < 0.001). Breech presentation is more strongly associated with non-macerated stillbirth (macerated: RR: 2.24, 95% CI: 1.26 to 4.00, *p* = 0.006; non-macerated: RR: 6.55, 95% CI: 4.16 to 10.31, *p* < 0.001), shoulder and face presentation remain similar across both classifications. Being a mother aged over 25 years was statistically associated with a higher risk of macerated stillbirth, yet a higher risk of non-macerated stillbirth was only present within the age groups 36 years and over. Similarly for distance travelled to the hospital a higher risk of macerated stillbirth was associated with distances travelled in categories 31-50 km, 51-100 km and > 100 km and higher risk of non-macerated stillbirth were only found in distance categories 10-30 km and 31-50 km (Fig. 2a-c and Supplementary table 3, supplementary file P17-19).

Further work was carried out exploring the association between birthweight and type of stillbirth outcome (macerated or non-macerated), with these findings suggesting that low birthweight < 2.3 kg was associated with a higher probability of a macerated stillbirth and higher birthweight > 3.0 kg was associated with a higher probability of non-macerated stillbirth (Supplementary Fig. 2, supplementary file P20). Similarly, low gestational age < 36 weeks was associated with a higher probability of macerated stillbirth and > 38 weeks gestational age was associated with a higher probability of non-macerated stillbirth (Supplementary Fig. 3, supplementary file P21).

## Discussion

This study identified it was possible to classify stillbirths using an established system at the MMSH. The feasibility of taking photographs of stillborn babies to improve the classification was met, it was socially and culturally acceptable to collect data. The primary research aims of this manuscript were to identify sociodemographic, living environment, and health status factors associated with stillbirths and the associations between pregnancy and birth factors and stillbirth.

This study identified several associations with stillbirth based on demographic, living environment and health status. Higher odds of stillbirth were associated with low levels of maternal education, further distance to travel from home to the hospital, living in a shack, maternal hypertension and having had a previous stillbirth after adjusting for all sociodemographic and health features. Pregnancy and birth related factors associated with higher odds of stillbirth included reported birthing complications, duration of labour being ≥ 18 h, antepartum haemorrhage, prolonged or obstructed labour, vaginal breech delivery emergency caesarean-section delivery, and signs of trauma to the neonate following birth. The data were suggestive that iron or folic acid supplements were associated with lower risk of stillbirth.

Pregnancy and birth related factors associated with higher risk of non-macerated stillbirth included shoulder presentation, compound presentation and breech presentation compared to cephalic presentation, the mother reporting as underweight compared to healthy weight and the use of traditional medicine. Factors associated with a higher risk of macerated stillbirth included, hypertension, a condition known to cause intrauterine growth restriction and fetal death, an event that most often occurs prior to labour, and maternal age (between 25–35 years). Ultrasound during pregnancy and the use of iron or folic acid supplements were associated with a lower risk of non-macerated stillbirth.

Data quality and completeness were excellent, a testament to the team based in MMSH. There were no refusals to participate, withdrawals or opt-outs which could be linked with multiple factors, women’s compliance, or a demonstration of the understanding for research in this area.

While we obtained signs of maceration, these were through observational reports and for the team in the UK, through photographs, this may mis-represent non-macerated stillbirth [15], thus unable to use the photographs as a robust method for determining time or cause of stillbirth. Study duration poses a limitation; we did not capture a full calendar year, rendering the stillbirth incidence only reflective of the time we conducted the study. This was a facility-based study, and whilst we are aware that many births occur in the community, and we did not collect the reason why a mother attended the hospital to deliver her baby, therefore, our sample may be prone to selection bias, this point is especially pertinent when considering the mothers who travelled a significant distance to attend hospital compared to those who lived within accessible distance. Finally, we did not capture maternal or early neonatal death to further support our findings.

We have explored the associations and predictive performance of our variables to generate hypotheses and inform future intervention targets. We have not aimed to develop a multivariable prediction model, nor have we quantitatively considered the role of confounding, as these were not the goals of the feasibility study. There was lower statistical power for the multinomial logistic regression analysis compared to the binary logistic regression, due to each outcome having fewer events.

Our research supported existing findings in relation to identifying determinants of stillbirth, including; low level of maternal education, history of previous stillbirth, maternal hypertension and prolonged or obstructed labour being associated with stillbirth [10, 16–19]. It is likely mothers with higher education levels are more aware of maternal health, therefore, the risk of stillbirth is higher among those with lower education levels, education is a determinant of socioeconomic status (SES), consistent with reports that low SES is associated with stillbirth [7]. A review conducted by Lamont et al. concludes that women who experience a stillbirth in their initial pregnancy have a higher risk of stillbirth in a subsequent pregnancy, stating risk of recurrent unexplained stillbirth is largely unstudied and evidence remains controversial [16].

Our findings on residential accommodation and toilet type and associations with stillbirth appear unique; research has previously been conducted on SES and associations with stillbirth, yet not at the level of detail we were able to explore in this study. Living in a shack compared to living in a house and having a pit latrine over a flushing toilet is evident of low SES.

It is well documented that non-cephalic fetal presentation increases risk of stillbirth [20], we explored fetal presentation in more detail; shoulder, compound and breech presentation were all associated independently with stillbirth. Cetin et al. report that the neglected shoulder presentation is now usually observed in LMICs, and is associated with increased risk of stillbirth and maternal morbidity and mortality [21]. Our findings on maternal age and associations with stillbirth were inconsistent with previous studies, we found higher odds as age increased from 25 years and above, but we did not identify an association between < 20 year-olds and stillbirth, despite reasonable representation within this age category. There is a known disparity between rural and urban geographic access to tertiary healthcare and a need to deploy strategies to enhance rural access [22, 23]. We found a clear correlation between an increased distance required to travel to hospital and higher odds of stillbirth. This finding should be interpreted with caution, as it is likely that only complicated deliveries attend MMSH from an extended distance due to the specialities in this hospital and clinical need, making those within this analysis at higher risk and this finding may be less related with the distance travelled and more related to the motivation underpinning the distance that mothers were willing to travel (i.e., the perceived risk of their pregnancy).

Bhusal et al. reports that in LMICs recent efforts have been made to classify stillbirth by the sign of maceration, with a distinction between macerated (antepartum) (death occurring > 12 h before birth) and non-macerated (intrapartum) (death occurring < 12 h before birth) stillbirth, suggesting macerated/antepartum stillbirth is influenced by maternal health and the quality of antenatal care and non-macerated/intrapartum stillbirth is assumed to reflect the availability and quality of intrapartum care.

This work has obtained baseline data on the factors influencing stillbirth in Kano, and in turn this will facilitate the development of improved public health interventions to reduce these preventable deaths and to improve maternal health. The next stage to make a change is to improve surveillance of stillbirth and to improve diagnostic capabilities within Kano, to provide more accurate information regarding cause of death and thus mitigate the causes of stillbirth. There are standardised guidelines for perinatal death audit, released by WHO in 2016 [24], the aim is to assist healthcare providers to establish effective systems to capture the number and causes of stillbirth. The guidelines set out clear steps for the identification of cases of stillbirth, data collection and data analysis. We recommend these guidelines are adopted by the healthcare team at MMSH. Improved data are needed so healthcare professionals can understand causes of stillbirths and take correct and focused action. We have reviewed some of the associations, but further focus is required on the causes.

The implications of the study are that it could be widely replicated in areas with similar problems or concerns, this research was conducted with extremely high levels of sensitivity due to the subject matter. The approaches taken were well received by mothers and healthcare workers and therefore this model of approach could easily be mirrored in similar settings with similar approaches to managing stillbirth.

Sufficient baseline data has been collected to identify at risk mothers (many of whom can be identified early in pregnancy), and can aim to improve their antenatal care, a programme to support mothers with previous experience of stillbirth could be implemented. Additionally, stillbirth education among the mothers and families with low levels of education and in the underserved areas could be rolled out. Supporting qualitative work found mothers were influenced by the family dynamic, and if elders and husbands disagreed with seeking medical support then it would be a challenge for labouring mothers to overcome, often presenting too late at the hospital to save the baby [25]. Demonstrating the need to deliver support-based or educational programmes at family and extended family level.

Preventing and controlling stillbirth is essential to achieve the target set by Every Newborn Action Plan in 2014 of 12 or fewer stillbirths per 1000 births in every country by 2030 [26]. To prevent stillbirth, understanding the gravity of the situation is paramount; this research has explored a way to determine an incidence of stillbirth in this particular setting and has identified many of the common associations with stillbirth and can inform future work and interventions. A large proportion of stillbirths are preventable, it is likely that high rates of stillbirth in this area is due to lack of access to antenatal and intrapartum care rather than lack of knowledge and education among healthcare workers. This is a hugely over-stretched hospital supporting an enormous population. Determinants of stillbirths are relatively amenable to intervention and a lot of work has been conducted globally, so the development of intervention with sufficient funding should be a relatively rapid process. Targeting at risk populations, recording all stillbirths are vital first steps required to start reducing the high burden of stillbirths in Kano.

## Supplementary Information

**Additional file 1.**

## Data Availability

The datasets analysed for this study can be provided on request. Please do contact the corresponding author, Rebecca Milton should you wish to request access to these data.
