# Supplementary material for: Incidence and sociodemographic, living environment and maternal health associations with stillbirth in a tertiary healthcare setting in Kano, Northern Nigeria
Source: BMC Pregnancy Childbirth. 2022 Sep 8;22:692. doi: 10.1186/s12884-022-04971-x (PMC9454147; doi:10.1186/s12884-022-04971-x)
Supplement: Supplementary file 1 — Additional file 1. [file 12884_2022_4971_MOESM1_ESM.docx]

**Supplementary Material**

Supplementary Data 1: Pre-Delivery Questionnaire – All enrolments.


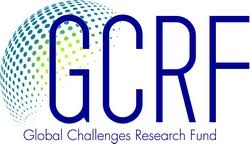

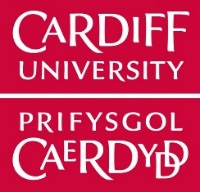


**A FEASIBILITY STUDY: STILLBIRTHS IN KANO**

**Pre Delivery Questionnaire – All Enrolments**

| **Number** | **Question** | **Answer options** | | **Guidance** |
| --- | --- | --- | --- | --- |
| 1 | Mother ID |  | | e.g. M001 |
| 2 | Researcher ID |  | | researcher’s initials |
| 3 | Date of enrolment |  | | DD/MM/YY |
| 4 | Time of enrolment |  | | time HH:MM (24-hour clock) |
| 5 | Mother’s age | <20 |  | please check (X) one option |
|  |  | 20-24 |  |  |
|  |  | 25-30 |  |  |
|  |  | 31-35 |  |  |
|  |  | 36-40 |  |  |
|  |  | >40 |  |  |
| 6 | Monthly household income (NGN) | <15,000 |  | please check (X) one option |
|  |  | 15,000 - 30,000 |  |  |
|  |  | 30,001 - 45,000 |  |  |
|  |  | 45,001 - 60,000 |  |  |
|  |  | 60,001 - 90,000 |  |  |
|  |  | 90,001 - 150,000 |  |  |
|  |  | 150,001 - 210,000 |  |  |
|  |  | >210,000 |  |  |
| 7 | Area of residence | Fagge |  | please check (X) one option |
|  |  | Municipal |  |  |
|  |  | Gwale |  |  |
|  |  | Nassarawa |  |  |
|  |  | G/Mallam |  |  |
|  |  | Kumbotso |  |  |
|  |  | Dala |  |  |
|  |  | Madobi |  |  |
|  |  | Kura |  |  |
|  |  | D/kuda |  |  |
|  |  | Warawa |  |  |
|  |  | Tarauni |  |  |
|  |  | Gezawa |  |  |
|  |  | Ungogo |  |  |
|  |  | Minjibir |  |  |
|  |  | Kunchi |  |  |
|  |  | Makoda |  |  |
|  |  | Danbatta |  |  |
|  |  | Gabasawa |  |  |
|  |  | Bichi |  |  |
|  |  | Tsanyawa |  |  |
|  |  | Bagwai |  |  |
|  |  | D/Tofa |  |  |
|  |  | Tofa |  |  |
|  |  | R/gado |  |  |
|  |  | Kabo |  |  |
|  |  | Gwarzo |  |  |
|  |  | Shanono |  |  |
|  |  | Kayaye |  |  |
|  |  | Rogo |  |  |
|  |  | Kiru |  |  |
|  |  | Bebeji |  |  |
|  |  | Bunkure |  |  |
|  |  | Rano |  |  |
|  |  | Kibiya |  |  |
|  |  | Garko |  |  |
|  |  | Wudil |  |  |
|  |  | Gaya |  |  |
|  |  | Ajingi |  |  |
|  |  | Albasu |  |  |
|  |  | T/wada |  |  |
|  |  | Sumaila |  |  |
|  |  | Takai |  |  |
|  |  | Doguwa |  |  |
|  |  | Other |  |  |
| *7a* | *If other please detail* |  | | please detail in free text format |
| 8 | Type of residence | Shack |  | please check (X) one option |
|  |  | House |  |  |
|  |  | Apartment |  |  |
|  |  | Other |  |  |
| *8a* | *If other please detail* |  | | please detail in free text format |
| 9 | Highest level of education | None |  | please check (X) one option |
|  |  | Primary |  |  |
|  |  | Secondary |  |  |
|  |  | College and above |  |  |
| 10 | Employment status | Unemployed |  | please check (X) one option |
|  |  | Employed |  |  |
| 11 | Access to water at home | Communal taps (stream or community well) |  | please check (X) one option |
|  |  | Municipal network (tap water) |  |  |
|  |  | Private borehole |  |  |
|  |  | Private well |  |  |
|  |  | Sachet water (pure water) |  |  |
|  |  | Water vendor (meruwa) |  |  |
| 12 | Toilet facilities at home | Squat with flush |  | please check (X) one option |
|  |  | Sit with flush |  |  |
|  |  | Pit latrine |  |  |
|  |  | Other |  |  |
| *12a* | *If other please detail* |  | | please detail in free text format |
| 13 | Number of previous pregnancies (including those which did not result in a live birth) |  | | numerical value (e.g. 5) |
| 14 | Number of other children |  | | numerical value (e.g. 4) |
| 15 | Ages of other children |  | | numerical values separated by commas (e.g. 2, 4, 7, 8) |
| 16 | Is the mother still breastfeeding another child? | Yes |  | please check (X) one option |
|  |  | No |  |  |
| *16a* | *If yes how old is the child being breastfed?* |  | | numerical value (months) (e.g. 24) |
| 17 | Has the mother had a stillbirth before? | Yes |  | please check (X) one option |
|  |  | No |  |  |
| *17a* | *If yes how many?* |  | | numerical value (e.g. 2) |
| 18 | Nutritional status *(mother’s perception)* | Underweight |  | please check (X) one option |
|  |  | Overweight |  |  |
|  |  | Healthy weight |  |  |
| 19 | Is the mother taking any medication? | Yes |  | please check (X) one option |
|  |  | No |  |  |
| *19a* | *Type of Medication* | Antibiotics |  | please check (X) all relevant options |
|  |  | Pain Relief |  |  |
|  |  | Other |  |  |
|  |  | Unknown |  |  |
| *19b* | *If yes, please detail* |  | | Please detail in free text, include product names if possible.  Examples :  ABX – amoxicillin  PR – paracetamol  O – malarone  [ABX-antibitoics, PR-pain relief, O-other, U-unknown] |
| 20 | Does the mother have any health conditions or concerns? | Yes |  | please check (X) one option |
|  |  | No |  |  |
| *20a* | *If yes please check all relevant options* | Malaria |  | please check (X) all relevant options  NB: This data is either self-reported or confirmed by the health practitioner, please mark **SR** for self-reported data |
|  |  | Typhoid |  |  |
|  |  | Cholera |  |  |
|  |  | Tuberculosis |  |  |
|  |  | HIV |  |  |
|  |  | Diabetes (gestational) |  |  |
|  |  | Diabetes (existing condition) |  |  |
|  |  | Sickle cell anaemia |  |  |
|  |  | Hypocalcaemia |  |  |
|  |  | Oedema / swelling |  |  |
|  |  | Respiratory problems |  |  |
|  |  | Hypertension |  |  |
|  |  | Hypotension |  |  |
|  |  | Stroke |  |  |
|  |  | Hyperemesis |  |  |
|  |  | Vaginal bleeding |  |  |
|  |  | Placental Abruption |  |  |
|  |  | Pre-eclampsia |  |  |
|  |  | Vesicovaginal Fistula (VVF) |  |  |
|  |  | Haemorrhage |  |  |
|  |  | Chorioamnionitis |  |  |
|  |  | Chest pain |  |  |
|  |  | Infection |  |  |
|  |  | Convulsions |  |  |
|  |  | Fibroid |  |  |
|  |  | Diarrhoea |  |  |
|  |  | Other |  |  |
| *20b* | *If other please detail* |  | | please detail in free text format |
| 21 | Did the mother receive antenatal care for this pregnancy? | Yes |  | please check (X) one option |
|  |  | No |  |  |
| *21a* | *If yes how many sessions did she attend?* |  | | numerical value (e.g. 4) |
| 22 | Has the mother noticed regular foetal movement in the past 24 hours? | Yes |  | please check (X) one option |
|  |  | No |  |  |
| *22a* | *If no when did she last notice foetal movement?* |  | | please detail in free text format |
| 23 | Has the mother had an ultra-scan during pregnancy? | Yes |  | please check (X) one option |
|  |  | No |  |  |
| *23a* | *If yes, what date was her last scan?* |  | | DD/MM/YY |
| 24 | Was the pin prick blood sample collected from the mother? | Yes |  | please check (X) one option |
|  |  | No |  |  |
| *24a* | *If no please detail reasons* |  | | please detail in free text format |
| *24b* | *If yes please record time the sample was taken* |  | | time MM:HH (24-hour clock) |

Supplementary Data 2: Post-Delivery Questionnaire Livebirth


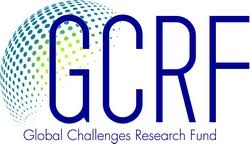

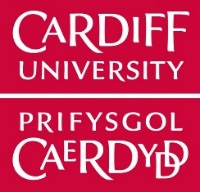


**A FEASIBILITY STUDY: STILLBIRTHS IN KANO**

**Post Delivery Questionnaire – Live Born Babies**

| **Number** | **Question** | **Answer options** | | **Guidance** |
| --- | --- | --- | --- | --- |
| 1 | Mother ID |  | | e.g., M001 |
| 2 | Researcher ID |  | | researcher’s initials |
| 3 | Baby ID |  | | e.g., NKLB001 |
| 4 | Date |  | | DD/MM/YY |
| 5 | Time of delivery |  | | time (HH:MM) 24-hour clock |
| 6 | Time QA completed |  | | time (HH:MM) 24-hour clock |
| 7 | Baby gender | Male |  | please check (X) one option |
|  |  | Female |  |  |
| 8 | Is this a singleton or multiple pregnancy? | Singleton |  | please check (X) one option |
|  |  | Multiple |  |  |
| *8a* | *If multiple how many babies?* |  | | numerical value (e.g. 2) |
| *8b* | *Please detail multiples and baby genders and IDs* |  | | Please report in free text format e.g., Triplets:  NKLB001 – female  NKSB001 – female  NKLB002 - male |
| 9 | Duration of ruptured membranes |  | | numerical value (hours) (e.g. 2) |
| 10 | Duration of labour |  | | numerical value (hours) (e.g. 9) |
| 11 | Were there any birthing complications | Yes |  | please check (X) one option |
|  |  | No |  |  |
| *11a* | *If yes* | Maternal pyrexia |  | please check (X) all relevant options |
|  |  | Shoulder dystocia |  |  |
|  |  | Cord complications (prolapse, tight around neck / body) |  |  |
|  |  | Other |  |  |
| *11b* | *If other please detail* |  | | please detail in free text format |
| 12 | Presentation of baby | Cephalic presentation |  | please check (X) one option |
|  |  | Breech presentation |  |  |
|  |  | Shoulder presentation |  |  |
|  |  | Compound presentation |  |  |
|  |  | Face presentation |  |  |
| 13 | Delivery of the baby | Spontaneous Vaginal Delivery |  | please check (X) all relevant options |
|  |  | Instrumental (forceps / ventouse) |  |  |
|  |  | Elective caesarean section |  |  |
|  |  | Emergency caesarean section |  |  |
|  |  | Vaginal breech delivery |  |  |
| 14 | External congenital abnormality | Yes |  | please check (X) one option |
|  |  | No |  |  |
| *14a* | *If yes please describe* |  | | please detail in free text format |
| 15 | Gestational age |  | | numerical value (weeks) (e.g. 37) |
| 16 | Birthweight |  | | numerical value (grams / kilograms) (please detail measurement used) (e.g. 1500g / 1.5kg) |
| 17 | Head circumference |  | | numerical value (centimetres) (e.g. 14) |
| 18 | Are there signs of trauma | Yes |  | please check (X) one option |
|  |  | No |  |  |
| *18a* | *If yes, please describe* |  | | please detail in free text format |
| 19 | APGAR Score |  | | numerical value (e.g. 4) |
| 20 | Signs of maternal infection | Yes |  | please check (X) one option |
|  |  | No |  |  |
| *20a* | *If yes, please describe* |  | | please detail in free text format |
| 21 | Signs of infant infection | *Yes*  *No* | |  |
| 21a | If yes, please describe |  | |  |

**Time Matched Control Cases Only**

| **Number** | **Question** | **Answer options** |  | **Guidance** |
| --- | --- | --- | --- | --- |
| 1 | Head swab taken | Yes |  | please check (X) one option |
|  |  | No |  |  |
| *1a* | *Time head swab was taken* |  | | Time (HH:MM) 24-hour clock |
| 2 | Ear swab taken | Yes |  | please check (X) one option |
|  |  | No |  |  |
| *2a* | *Time ear swab taken* |  | | Time (HH:MM) 24-hour clock |
| 3 | Photograph taken | Yes |  | please check (X) one option |
|  |  | No |  |  |
| *3a* | *Time photograph taken* |  | | Time (HH:MM) 24-hour clock |
| 4 | Umbilical cord blood extracted? | Yes |  | please check (X) one option |
|  |  | No |  |  |
| *4a* | *If not, would it have been possible? i.e. blood present?* | Yes |  | please check (X) one option |
|  |  | No |  |  |
| *4b* | *Time umbilical cord blood extracted* |  | | Time (HH:MM) 24-hour clock |

Supplementary Data 3: Post-Delivery Questionnaire Stillbirth


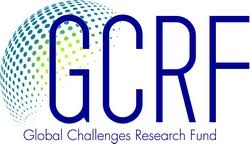

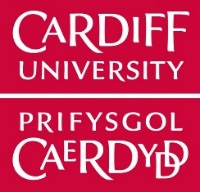


**A FEASIBILITY STUDY: STILLBIRTHS IN KANO**

**Post Delivery Questionnaire – Stillborn Babies**

| **Number** | **Question** | **Answer options** | | **Guidance** |
| --- | --- | --- | --- | --- |
| 1 | Mother ID |  | | e.g., M001 |
| 2 | Researcher ID |  | | Researchers initials |
| 3 | Baby ID |  | |  |
| 4 | Date |  | | DD/MM/YY |
| 5 | Time of delivery |  | | time (HH:MM) 24-hour clock |
| 6 | Time QA completed |  | | time (HH:MM) 24-hour clock |
| 7 | Baby gender | Male |  | please check (X) one option |
|  |  | Female |  |  |
|  |  | Unable to determine |  |  |
| 8 | Is this a singleton or multiple pregnancy? | Singleton |  | please check (X) one option |
|  |  | Multiple |  |  |
| *8a* | *If multiple how many babies?* |  | | numerical value (e.g., 2) |
| *8b* | *Please detail multiples and baby genders and IDs* |  | | Please report in free text format e.g., Triplets:  NKLB001 – female  NKSB001 – female  NKLB002 - male |
| 9 | Duration of ruptured membranes |  | | numerical value (hours) (e.g., 2) |
| 10 | Duration of labour |  | | numerical value (hours) (e.g., 9) |
| 11 | Was the baby alive in-utero prior to delivery? | Yes |  | please check (X) one option |
|  |  | No |  |  |
|  |  | Unsure |  |  |
| 12 | Were there any birthing complications | Yes |  | please check (X) one option |
|  |  | No |  |  |
| *12a* | *If yes* | Maternal pyrexia |  | please check (X) all relevant options |
|  |  | Shoulder dystocia |  |  |
|  |  | Cord complications (prolapse, tight around neck / body) |  |  |
|  |  | Other |  |  |
| *12b* | *If other please detail* |  | | please detail in free text format |
| 13 | Presentation of baby | Cephalic presentation |  | please check (X) one option |
|  |  | Breech presentation |  |  |
|  |  | Shoulder presentation |  |  |
|  |  | Compound presentation |  |  |
|  |  | Face presentation |  |  |
| 14 | Delivery of the baby | Spontaneous Vaginal Delivery |  | please check (X) all relevant options |
|  |  | Instrumental (forceps / ventouse) |  |  |
|  |  | Elective caesarean section |  |  |
|  |  | Emergency caesarean section |  |  |
|  |  | Vaginal breech delivery |  |  |
| 15 | External congenital abnormality | Yes |  | please check (X) one option |
|  |  | No |  |  |
| *15a* | *If yes please describe* |  | | please detail in free text format |
| 16 | Gestational age |  | | numerical value (weeks) (e.g., 37) |
| 17 | Birth weight |  | | numerical value (grams / kilograms) (please detail measurement used) (e.g., 1500g / 1.5kg) |
| 18 | Head circumference |  | | numerical value (centimetres) (e.g. 14) |
| 19 | Stillbirth classification | Congenital anomaly |  | please check (X) one option |
|  |  | Suspected infection |  |  |
|  |  | Asphyxia |  |  |
|  |  | Prematurity |  |  |
|  |  | Unknown |  |  |
|  |  | Other |  |  |
| *19a* | *Other* |  | | please detail in free text format |
| 20 | Are there signs of trauma | Yes |  | please check (X) one option |
|  |  | No |  |  |
| *20a* | *If yes, please describe* |  | | please detail in free text format |
| 21 | Infant odour offensive | Yes |  | please check (X) one option |
|  |  | No |  |  |
| 22 | Maternal odour offensive | Yes |  | please check (X) one option |
|  |  | No |  |  |
| 23 | Signs of maternal infection | Yes |  | please check (X) one option |
|  |  | No |  |  |
| *23a* | *If yes, please describe* |  | | please detail in free text format |
| 24 | Signs of infant infection | Yes |  | please check (X) one option |
|  |  | No |  |  |
| *24a* | *If yes, please describe* |  | | please detail in free text format |
| 25 | Head swab taken | Yes |  | please check (X) one option |
|  |  | No |  |  |
| *25a* | *Time head swab was taken* |  |  | time (HH:MM) 24-hour clock |
| 26 | Ear swab taken | Yes |  | please check (X) one option |
|  |  | No |  |  |
| *26a* | *Time ear swab was taken* |  |  | time (HH:MM) 24-hour clock |
| 27 | Photograph taken | Yes |  | please check (X) one option |
|  |  | No |  |  |
| *27a* | *Time photograph was taken* |  |  | time (HH:MM) 24-hour clock |
| 28 | Umbilical cord blood extracted? | Yes |  | please check (X) one option |
|  |  | No |  |  |
| *28a* | *If not, would it have been possible? i.e. blood present?* | Yes |  | please check (X) one option |
|  |  | No |  |  |
| *28b* | *Time umbilical cord blood was extracted* |  | | time (HH:MM) 24-hour clock |
| 29 | Comment box |  | | Please include any other information you feel is relevant to the stillbirth. |

Supplementary Table 1: Maternal characteristics

|  | | Total | |
| --- | --- | --- | --- |
|  |  | Count | Column n % |
| Mother's age in years (n=1926) | <20 | 186 | 9.7 |
|  | 20-24 | 566 | 29.4 |
|  | 25-30 | 656 | 34.1 |
|  | 31-35 | 258 | 13.4 |
|  | 36-40 | 209 | 10.9 |
|  | >40 | 51 | 2.6 |
| Monthly household income (n=1926) | Up to 30,000 | 560 | 29.1 |
|  | 30,001 - 45,000 | 630 | 32.7 |
|  | 45,001 - 60,000 | 521 | 27.1 |
|  | 60,001 - 90,000 | 178 | 9.2 |
|  | 90,001 - 150,000 | 37 | 1.9 |
| Type of residence (n=1926) | Apartment | 581 | 30.2 |
|  | House | 907 | 47.1 |
|  | Shack | 437 | 22.7 |
|  | Other | 1 | 0.1 |
| Highest level of education (n=1926) | None | 440 | 22.8 |
|  | Primary | 298 | 15.5 |
|  | Secondary | 990 | 51.4 |
|  | College and above | 198 | 10.3 |
| Employment Status (n=1926) | Employed | 860 | 44.7 |
|  | Unemployed | 1066 | 55.3 |
| Access to water at home (n=1926) | Communal taps | 250 | 13.0 |
|  | Municipal network | 366 | 19.0 |
|  | Private borehole | 109 | 5.7 |
|  | Private well | 565 | 29.3 |
|  | Water vendor (incl. sachet) | 636 | 33.0 |
| Toilet facilities at home (n=1926) | Squat with flush | 1142 | 59.3 |
|  | Sit with flush | 175 | 9.1 |
|  | Pit Latrine | 604 | 31.4 |
|  | Other | 5 | 0.3 |
| Pregnancy history (n=1926) | Been pregnant before | 1476 | 76.6 |
|  | Not been pregnant before | 450 | 23.4 |
| Previous stillbirth (n=1926) | No | 1636 | 84.9 |
|  | Yes | 290 | 15.1 |
| Grand-multiparity (n=1926) | Yes | 698 | 36.2 |
| Nutritional status (mothers’ perception) (n=1926) | Healthy Weight | 1715 | 89.0 |
|  | Overweight | 109 | 5.7 |
|  | Underweight | 102 | 5.3 |
| Medication (n=1926) | Unknown | 1 | 0.1 |
|  | No | 501 | 26.0 |
|  | Yes | 1424 | 73.9 |
| Health conditions (n=1926) | Yes | 1036 | 53.8 |
|  | No | 890 | 46.2 |
| Antibiotics (n=1926) | No | 1776 | 92.2 |
|  | Yes | 150 | 7.8 |
| Antimalarials (n=1926) | No | 1384 | 71.9 |
|  | Yes | 542 | 28.1 |
| Antihypertensives (n=1926) | No | 1852 | 96.2 |
|  | Yes | 74 | 3.8 |
| Anti-inflammatories (n=1926) | No | 1922 | 99.8 |
|  | Yes | 4 | 0.2 |
| Vitamins (n=1926) | No | 1770 | 91.9 |
|  | Yes | 156 | 8.1 |
| Folic acid / iron / platelets (n=1926) | No | 1147 | 59.6 |
|  | Yes | 779 | 40.4 |
| Pain relief (n=1926) | No | 864 | 44.9 |
|  | Yes | 1062 | 55.1 |
| Traditional medication (n=1926) | No | 1662 | 86.3 |
|  | Yes | 264 | 13.7 |
| ART medication (n=1926) | No | 1921 | 99.7 |
|  | Yes | 5 | 0.3 |
| Other medications (n=1926) | No | 1809 | 93.9 |
|  | Yes | 117 | 6.1 |
| Malaria (n=1926) | No | 1355 | 70.4 |
|  | Yes | 571 | 29.6 |
| Typhoid (n=1926) | No | 1903 | 98.8 |
|  | Yes | 23 | 1.2 |
| Hypertension (n=1926) | No | 1738 | 90.2 |
|  | Yes | 188 | 9.8 |
| Driving time from home to hospital (n=1925) | <10km | 1125 | 58.4 |
|  | 10-30km | 696 | 36.1 |
|  | 31-50km | 66 | 3.4 |
|  | 51-100km | 22 | 1.1 |
|  | >100km | 16 | 0.8 |
| Signs of maternal infection (n=1926) | Yes | 41 | 2.1 |
|  | No | 1885 | 97.9 |
| Has regular foetal movement in the last 24 hours (n=1926) | Yes | 1808 | 93.9 |
|  | No | 118 | 6.1 |
| Ultrasound during this pregnancy? (n=1926) | Yes | 1420 | 73.7 |
|  | No | 506 | 26.3 |
| Baby gender (n=1996) | Male | 1086 | 54.4 |
|  | Female | 908 | 45.5 |
|  | Unable to determine | 2 | 0.1 |
| Presentation of the baby (n=1998) | Shoulder presentation | 10 | 0.5 |
|  | Face presentation | 13 | 0.7 |
|  | Compound presentation | 23 | 1.2 |
|  | Cephalic presentation | 1767 | 88.4 |
|  | Breech presentation | 185 | 9.3 |
| Mode of delivery (n=1998) | Vaginal breech delivery | 42 | 2.1 |
|  | SVD | 1646 | 82.4 |
|  | Instrumental | 1 | 0.1 |
|  | Emergency C-section | 223 | 11.2 |
|  | Elective C-section | 86 | 4.3 |
| Signs of trauma (n=1998) | Yes | 100 | 5.0 |
|  | No | 1898 | 95.0 |
| Single or multiple pregnancy (n=1926) | Singleton | 1857 | 96.4 |
|  | Multiple | 69 | 3.6 |

Supplementary Table 2: Pseudo r^2^ for the multivariable model include demographic features

|  |  |  | **AUROC** | | | **Pseudo r-squared** | | |
| --- | --- | --- | --- | --- | --- | --- | --- | --- |
| **Block** | **N parameters** | **EPV** | **Value** | **Abs diff** | **%diff** | **Value** | **Abs diff** | **%diff** |
| 1 | 9 | 23 | 0.675 |  |  | 0.0562 |  |  |
| 2 | 16 | 13 | 0.719 | 0.044 | 6.5 | 0.0992 | 0.043 | 76.5 |
| 3 | 29 | 7 | 0.753 | 0.034 | 4.7 | 0.1223 | 0.0231 | 23.3 |
| 4 | 31 | 7 | 0.768 | 0.015 | 2.0 | 0.1394 | 0.0171 | 14.0 |

**

Supplementary Figure 1: Predicted probability of stillbirth based on birthweight

Supplementary Table 3: Associations between sociodemographic, living environment, maternal health and medical history, pregnancy history and pregnancy/birth related variables and macerated and non-macerated stillbirths

| **Domain** | **Variable** | **Categories** | **Stillbirth overall** | | **Macerated stillbirth** | | **Non-macerated stillbirth** | | **Macerated vs. non-macerated** |
| --- | --- | --- | --- | --- | --- | --- | --- | --- | --- |
|  |  |  | **OR (95% CI)** | **p-value** | **RR (95% CI)** | **p-value** | **RR (95% CI)** | **p-value** | **p-value** |
| Demographics | Mothers age (years) | <20 | 1.34 (0.73 to 2.47) | 0.349 | 0.95 (0.30 to 2.96) | 0.933 | 1.55 (0.76 to 3.16) | 0.230 | 0.027 |
|  |  | 20-24 | Reference category | | | | | | |
|  |  | 25-30 | 1.96 (1.31 to 2.95) | 0.001 | 3.07 (1.64 to 5.77) | <0.001 | 1.36 (0.80 to 2.31) | 0.252 |  |
|  |  | 31-35 | 2.19 (1.35 to 3.57) | 0.002 | 4.10 (2.04 to 8.24) | <0.001 | 1.16 (0.57 to 2.36) | 0.683 |  |
|  |  | 36-40 | 2.34 (1.41 to 3.89) | 0.001 | 2.66 (1.19 to 5.93) | 0.017 | 2.16 (1.15 to 4.07) | 0.017 |  |
|  |  | >40 | 3.68 (1.76 to 7.72) | 0.001 | 3.81 (1.19 to 12.18) | 0.024 | 3.61 (1.47 to 8.85) | 0.005 |  |
|  | Monthly household income (NGN) | 45,001 or higher | Reference category | | | | | | |
|  |  | Up to 45,000 | 1.22 (0.90 to 1.65) | 0.195 | 1.5 (0.97 to 2.32) | 0.071 | 1.02 (0.69 to 1.52) | 0.911 | 0.196 |
|  | Employment status | Employed | Reference category | | | | | | |
|  |  | Unemployed | 1.07 (0.80 to 1.43) | 0.633 | 0.83 (0.55 to 1.24) | 0.357 | 1.37 (0.92 to 2.04) | 0.120 | 0.072 |
|  | Highest level of education | Secondary school or above | Reference category | | | | | | |
|  |  | None or primary school | 3.40 (2.52 to 4.59) | <0.001 | 3.63 (2.36 to 5.56) | <0.001 | 3.21 (2.14 to 4.80) | <0.001 | 0.673 |
| Living environment | Distance travelled to the hospital | <10km | Reference category | | | | | | |
|  |  | 10-30km | 1.57 (1.15 to 2.14) | 0.005 | 1.46 (0.93 to 2.29) | 0.099 | 1.66 (1.10 to 2.51) | 0.016 | 0.448 |
|  |  | 31-50km | 5.43 (3.14 to 9.39) | <0.001 | 5.75 (2.79 to 11.83) | <0.001 | 5.15 (2.51 to 10.53) | <0.001 |  |
|  |  | 51-100km | 5.18 (2.08 to 12.92) | <0.001 | 7.83 (2.74 to 22.37) | <0.001 | 2.81 (0.63 to 12.56) | 0.177 |  |
|  |  | >100km | 5.38 (1.83 to 15.83) | 0.002 | 9.12 (2.79 to 29.79) | <0.001 | 2.04 (0.26 to 16.14) | 0.499 |  |
|  | Type of residence | Apartment or house | Reference category | | | | | | |
|  |  | Shack or other | 2.59 (1.92 to 3.49) | <0.001 | 2.26 (1.48 to 3.45) | <0.001 | 2.92 (1.96 to 4.33) | <0.001 | 0.368 |
|  | Primary household water source | Municipal network | Reference category | | | | | | |
|  |  | Not municipal network | 1.90 (1.22 to 3.96) | 0.004 | 2.00 (1.06 to 3.77) | 0.033 | 1.82 (1.01 to 3.29) | 0.047 | 0.834 |
|  | Household toilet facilities | Toilet with flush | Reference category | | | | | | |
|  |  | Pit latrine or other | 2.94 (2.20 to 3.94) | <0.001 | 3.12 (2.08 to 4.70) | <0.001 | 2.79 (1.89 to 4.12) | <0.001 | 0.686 |
| Health and medical history | Self-perceived nutritional status | Healthy Weight | Reference category | | | | | | |
|  |  | Overweight | 0.97 (0.51 to 1.84) | 0.918 | 1.07 (0.46 to 2.50) | 0.882 | 0.87 (0.35 to 2.19) | 0.766 | 0.246 |
|  |  | Underweight | 1.97 (1.19 to 3.28) | 0.009 | 1.20 (0.51 to 2.81) | 0.681 | 2.73 (1.50 to 4.98) | 0.001 |  |
|  | Medication | Antibiotics | 1.57 (0.98 to 2.50) | 0.061 | 1.41 (0.71 to 2.77) | 0.324 | 1.71 (0.94 to 3.14) | 0.081 | 0.657 |
|  |  | Anti-inflammatories | 2.15 (0.24 to 19.29) | 0.496 | N/A | N/A | 4.13 (0.46 to 37.26) | 0.207 | 0.988 |
|  |  | Pain relief | 0.65 (0.49 to 0.87) | 0.003 | 0.65 (0.44 to 0.98) | 0.038 | 0.65 (0.44 to 0.96) | 0.029 | 0.985 |
|  |  | Traditional | 1.35 (0.92 to 1.98) | 0.126 | 1.06 (0.59 to 1.89) | 0.855 | 1.64 (1.01 to 2.67) | 0.047 | 0.239 |
|  |  | ART | 2.15 (0.24 to 19.29) | 0.496 | 4.52 (0.50 to 40.73) | 0.179 | N/A | N/A | 0.980 |
|  |  | Other | 0.86 (0.46 to 1.64) | 0.655 | 0.65 (0.23 to 1.80) | 0.405 | 1.07 (0.48 to 2.35) | 0.870 | 0.438 |
|  | Supplements | Vitamins | 1.03 (0.61 to 1.74) | 0.921 | 1.01 (0.48 to 2.12) | 0.982 | 1.04 (0.52 to 2.11) | 0.905 | 0.946 |
|  |  | Folic acid / iron / haemoglobin | 0.65 (0.48 to 0.88) | 0.006 | 0.66 (0.43 to 1.02) | 0.059 | 0.64 (0.42 to 0.97) | 0.033 | 0.900 |
|  | Health conditions | Malaria | 0.66 (0.47 to 0.93) | 0.018 | 0.67 (0.41 to 1.07) | 0.094 | 0.66 (0.42 to 1.05) | 0.079 | 0.991 |
|  |  | Fever / Infection | 2.07 (0.99 to 4.35) | 0.054 | 1.92 (0.67 to 5.49) | 0.224 | 2.22 (0.85 to 5.75) | 0.102 | 0.835 |
|  |  | Hypertension | 1.88 (1.26 to 2.82) | 0.002 | 2.20 (1.29 to 3.76) | 0.004 | 1.60 (0.91 to 2.83) | 0.104 | 0.402 |
|  | Pregnancy history | Previous stillbirth | 2.96 (2.14 to 4.08) | <0.001 | 5.28 (2.61 to 10.66) | <0.001 | 3.15 (1.75 to 5.66) | <0.001 | 0.420 |
|  |  | Grand Multiparity (>5 previous pregnancies) | 2.43 (1.82 to 3.24) | <0.001 | 3.11 (2.06 to 4.71) | <0.001 | 1.94 (1.32 to 2.87) | 0.001 | 0.094 |
| Pregnancy / Birth Related | Singleton or multiple pregnancy | Singleton | Reference category | | | | | | |
|  |  | Multiple | 0.51 (0.25 to 1.05) | 0.068 | 0.53 (0.19 to 1.47) | 0.223 | 0.49 (0.18 to 1.34) | 0.163 | 0.901 |
|  | Ultrasound scan during pregnancy | No | Reference category | | | | | | |
|  |  | Yes | 0.74 (0.54 to 1.01) | 0.054 | 0.83 (0.53 to 1.30) | 0.420 | 0.66 (0.44 to 1.00) | 0.049 | 0.443 |
|  | Presentation of baby on delivery | Cephalic | Reference category | | | | | | |
|  |  | Shoulder | 17.17 (4.79 to 61.54) | <0.001 | 15.23 (3.35 to 69.22) | <0.001 | 19.66 (4.31 to 89.72) | <0.001 | 0.009 |
|  |  | Face | 2.08 (0.46 to 9.48) | 0.344 | 1.85 (0.24 to 14.48) | 0.559 | 2.38 (0.30 to 18.75) | 0.409 |  |
|  |  | Compound | 8.80 (3.79 to 20.43) | <0.001 | 1.56 (0.20 to 12.09) | 0.669 | 18.15 (7.47 to 44.05) | <0.001 |  |
|  |  | Breech | 4.12 (2.85 to 5.96) | <0.001 | 2.24 (1.26 to 4.00) | 0.006 | 6.55 (4.16 to 10.31) | <0.001 |  |
|  | Birthing complications | No | Reference category | | | | | | |
|  |  | Yes | 5.98 (4.43 to 8.07) | <0.001 | 4.88 (3.24 to 7.37) | <0.001 | 7.23 (4.84 to 10.80) | <0.001 | 0.160 |
|  | Duration of ROM | < 18 hours | Reference category | | | | | | |
|  |  | 18 hours or longer | 2.92 (2.10 to 4.07) | <0.001 | 2.34 (1.45 to 3.77) | <0.001 | 3.52 (2.30 to 5.40) | <0.001 | 0.413 |
|  |  | Unknown | 3.42 (1.59 to 7.36) | 0.002 | 2.97 (1.02 to 8.68) | 0.046 | 3.88 (1.46 to 10.33) | 0.007 |  |
|  | Antepartum haemorrhage | No | Reference category | | | | | | |
|  |  | Yes | 6.66 (4.59 to 9.68) | <0.001 | 8.00 (4.96 to 12.88) | <0.001 | 5.55 (3.39 to 9.10) | <0.001 | 0.248 |
|  | Delivery of baby | Spontaneous vaginal delivery | Reference category | | | | | | |
|  |  | Vaginal breech delivery | 4.61 (2.34 to 9.05) | <0.001 | 3.59 (1.35 to 9.55) | 0.010 | 5.59 (2.47 to 12.66) | <0.001 | 0.945 |
|  |  | Emergency C-section | 2.89 (2.02 to 4.15) | <0.001 | 2.63 (1.59 to 4.35) | <0.001 | 3.14 (1.97 to 5.02) | <0.001 |  |
|  |  | Elective C-section | 0.12 (0.02 to 0.87) | 0.036 | 0.25 (0.03 to 1.79) | 0.165 | Not estimable | |  |
|  | Prolonged obstructive labour | No | Reference category | | | | | | |
|  |  | Yes | 3.34 (1.96 to 5.69) | <0.001 | 2.01 (0.84 to 4.79) | 0.114 | 4.65 (2.49 to 8.65) | <0.001 | 0.100 |
|  | Sex | Male | Reference category | | | | | | |
|  |  | Female | 1.02 (0.76 to 1.36) | 0.913 | 1.22 (0.82 to 1.83) | 0.623 | 0.86 (0.58 to 1.27) | 0.740 | 0.444 |
|  |  | Unable to determine | Not estimable | | | | | | |
|  | Gestational age (weeks) | Linear term | 0.23 (0.11 to 0.49) | <0.001 | 0.24 (0.09 to 0.65) | 0.005 | 0.24 (0.10 to 0.60) | 0.002 |  |
|  |  | Squared term | 1.02 (1.01 to 1.03) | 0.001 | 1.02 (1.00 to 1.03) | 0.018 | 1.02 (1.01 to 1.03) | 0.006 | 0.481 |
|  | Birthweight (kg) | Spline term 1 | 0.22 (0.16 to 0.30) | <0.001 | 0.20 (0.13 to 0.29) | <0.001 | 0.26 (0.17 to 0.38) | <0.001 |  |
|  |  | Spline term 2 | 5.67 (3.59 to 8.96) | <0.001 | 5.61 (2.97 to 10.58) | <0.001 | 5.51 (3.09 to 9.81) | <0.001 | 0.280 |

*Base outcome = livebirth.

Supplementary Figure 2: Association between birthweight and type of stillbirth

Supplementary Figure 3: Association between gestational age and type of stillbirth
